# Editorial: Behavior change approaches to improve children and families' dietary intake and 24h movement behaviors

**DOI:** 10.3389/fpubh.2023.1211601

**Published:** 2023-06-09

**Authors:** Brittany J. Johnson, Katherine L. Downing, Paul Chadwick, Jessica S. Gubbels

**Affiliations:** ^1^Caring Futures Institute, Flinders University, Adelaide, SA, Australia; ^2^Institute for Physical Activity and Nutrition, School of Exercise and Nutrition Sciences, Deakin University, Geelong, VIC, Australia; ^3^Centre for Behaviour Change, University College London, London, United Kingdom; ^4^Department of Health Promotion, School of Nutrition and Translational Research in Metabolism, Faculty of Health, Medicine and Life Sciences, Maastricht University, Maastricht, Netherlands

**Keywords:** behavior change, families, nutrition, movement, intervention design

Parents and caregivers play a key role in shaping the development of children's dietary habits and 24-h movement behaviors (1, 2). Supporting parents/caregivers to increase children's healthy food intake and physical activity, reduce nutrient-poor food intake and sedentary time, and improve sleep, all require behavior change. Changing behavior is complex, and further complicated when targeting parents/caregivers to improve children's health outcomes indirectly (3). There are numerous factors that may influence the foods and movement opportunities parents/caregivers provide to their children. To effectively intervene and support families, it is crucial to first determine what is needed to help change parental behaviors. Behavior change theories provide frameworks [e.g., (4, 5)] to help untangle the complexity of behavior change. However, the rationale and theoretical underpinning of behavior change approaches is often unclear in the reporting of interventions to support children and families (6, 7).

Due to the varied application, evaluation and reporting of behavior change approaches, we have a limited understanding of the most effective methods to support children and families in changing diet and movement behaviors, and which approaches are suitable in different settings and populations. There remains a need for high-quality research to explore the influences of behavior change approaches and to test their effectiveness of behavior change approaches in interventions, in a variety of settings and with underserved populations.

This Research Topic aimed to present the latest evidence exploring and testing behavior changes approaches to support children and families to improve diet intake and movement behaviors, and includes four manuscripts from across the globe.

Roba et al. examined the associations between antenatal care utilization and nutrition counseling with pregnant women's knowledge about infant and young child feeding. This study was set in rural and semi-urban areas in Eastern Ethiopia. Understanding predictors of expecting mother's knowledge can inform the design of appropriate interventions to integrate into existing antenatal services. The study showed that antenatal care utilization and nutrition counseling during pregnancy are indeed significantly associated with women's knowledge, while distance to health facilities was negatively associated with knowledge. This indicates the importance of proper antenatal counseling for young children's feeding.

Macchi et al. present findings from a multi-site randomized controlled trial testing receipt of weekly SMS message to improve caregiver feeding practices, to impact infant dietary intake, in Puerto Rico and Hawaii. This study included participants of the WIC (Women, Infants, and Children) program using a brief intervention, suitable for low-cost delivery at scale. Macchi et al. acknowledged the complexities of intervention design including the timing of intervention delivery for the behavior/s of interest, and consideration of intervention dose and inclusion of other caregivers involved in infant feeding. Such complexities are supported by existing literature in children's health behaviors, for example intervention dose (8).

Marshall et al. applied intervention coding methods from the TOPCHILD Collaboration (9) to compare components in the INFANT (INfant Feeding, Active play, and NuTrition) program from the original randomized controlled trial first evaluated in the late 2000's (10), with the current version being tested in state-wide scale up trial in Victoria, Australia (11). INFANT is an early life nutrition and movement behavior intervention seeking to impact both parent and child outcomes, and one of the few delivered at scale. This systematic approach to retrospectively code and describe intervention components, along with the rationale for changes through the scale-up process, provide a unique case study in understanding the behavior change potential in an intervention to change both parent and child health behaviors.

Rainham et al. present a perspective article proposing the benefits of targeting both parents and children to be more active together to addressing physical inactivity and sedentary behaviors. This perspective argues for a greater focus on addressing the home and social environments including key influences on children's behavior (e.g., parents, siblings, and friends). There are opportunities to further explore the potential influence of intrafamilial behaviors, while paying close attention to unintended consequences of efforts to change one health behavior (e.g., dietary intake) on other related behaviors (e.g., sedentary behavior) (12, 13).

In conclusion, this Research Topic presents recent explorations into behavior change and assists in the transparent publication of different behavior change approaches to support families promote nutrition and movement behaviors. This Research Topic touches on some of the challenges faced when addressing family heath behavior change, including (1) multiple layers of target participants (parents and children) and target behaviors as highlighted in Marshall et al. and Rainham et al., (2) designing accessible supports for underserved populations as targeted in Roba et al. and Macchi et al., and (3) approaches for intervention delivery at scale in Macchi et al. and Marshall et al. Continued research in this area will help to collectively advance the integration of behavioral medicine in the field of nutrition and 24h movement behaviors.

## Author contributions

BJ drafted the editorial. JG, KD, and PC critically edited the editorial. All authors were guest associate editors of the Research Topic. All authors contributed to the article and approved the submitted version.
